# Evolutionary relationships and diversification of *barhl *genes within retinal cell lineages

**DOI:** 10.1186/1471-2148-11-340

**Published:** 2011-11-21

**Authors:** Laura-Nadine Schuhmacher, Shahad Albadri, Mirana Ramialison, Lucia Poggi

**Affiliations:** 1 Centre for Organismal Studies, University of Heidelberg, Heidelberg 69120, Germany; 2 The Victor Chang Cardiac Research Institute, Darlinghurst NSW 2010, Australia

## Abstract

**Background:**

Basic helix-loop-helix and homeodomain transcription factors have been shown to specify all different neuronal cell subtypes composing the vertebrate retina. The appearance of gene paralogs of such retina-specific transcription factors in lower vertebrates, with differently evolved function and/or conserved non-coding elements, might provide an important source for the generation of neuronal diversity within the vertebrate retinal architecture. In line with this hypothesis, we investigated the evolution of the homeobox Barhl family of transcription factors, *barhl1 *and *barhl2*, in the teleost and tetrapod lineages. In tetrapod *barhl2*, but not *barhl1*, is expressed in the retina and is important for amacrine cell specification. Zebrafish has three *barhl *paralogs: *barhl1.1, barhl1.2 *and *barhl2*, but their precise spatio-temporal retinal expression, as well as their function is yet unknown.

**Results:**

Here we performed a meticulous expression pattern comparison of all known *barhl *fish paralogs and described a novel *barhl *paralog in medaka. Our detailed analysis of zebrafish *barhl *gene expression in wild type and mutant retinas revealed that only *barhl1.2 *and *barhl2 *are present in the retina. We also showed that these two paralogs are expressed in distinct neuronal lineages and are differently regulated by Atoh7, a key retinal-specific transcription factor. Finally, we found that the two retained medaka fish *barhl *paralogs, *barhl1 *and *barhl2*, are both expressed in the retina, in a pattern reminiscent of zebrafish *barhl1.2 *and *barhl2 *respectively. By performing phylogenetic and synteny analysis, we provide evidence that *barhl *retinal expression domain is an ancestral feature, probably lost in tetrapods due to functional redundancy.

**Conclusions:**

Functional differences among retained paralogs of key retina-specific transcription factors between teleosts and tetrapods might provide important clues for understanding their potential impact on the generation of retinal neuronal diversity. Intriguingly, within teleosts, retention of zebrafish *barhl1.2 *and its medaka ortholog *barhl1 *appears to correlate with the acquisition of distinct signalling mechanisms by the two genes within distinct retinal cell lineages. Our findings provide a starting point for the study of *barhl *gene evolution in relation to the generation of cell diversity in the vertebrate retina.

## Background

The vertebrate retina is organized into a complex network of cell layers, namely the ganglion cell layer (GCL) which contains retinal ganglion cells (RGCs) and displaced amacrine cells (ACs), the inner nuclear layer (INL) which consists of ACs, horizontal, bipolar and Müller glia cells, and the outer nuclear layer (ONL) which is made up of cone and rod photoreceptors. This strikingly complex architectural plan of the retina is extremely well conserved across vertebrate species, probably in direct correlation with the conservation of the key regulatory factors that govern retinal development. Several members of the basic helix-loop-helix (bHLH) and homeodomain family of transcription factors are known to play a role in the determination of retinal progenitor competence and cell fate, a function that is highly conserved from fish to mammals [1]. Much less is known on the contribution of different functional paralogs of retina-specific transcription factors, which arose subsequently to rounds of whole genome duplication (WGD) during vertebrate evolution [2]. Indeed, it has been proposed that after WGD, duplicated genes can either accumulate loss-of-function mutations and are functionally lost (non-functionalization [3,4]) or acquire a new function (neo-functionalization), or split the ancestral function between the paralogs (sub-functionalization) [2]), therefore adding complexity to the developmental gene network that shapes organ formation. The genes of the *barhl *family encoding the homeobox transcription factors Barhl1 and Barhl2, have been shown to be expressed in more or less overlapping domains of the central nervous system and have partially redundant functions in neural subtype cell identity, migration and survival [5,6]; however, *barhl2 *members appear to be uniquely expressed in the retina [7,8]. In particular, Barhl2 is a pan-vertebrate regulator of the specification and survival of ACs and RGCs [9-11]. Forced expression of Barhl2 in the mouse retina promotes the differentiation of glycinergic amacrine cells at the expense of bipolar and Müller cells [10]. Additionally, analysis of Barhl2-null retinas suggests that Barhl2 plays a critical role in both AC subtype determination and in RGC survival [9]. The *Xenopus *Barhl2 ortholog (previously named Xbh1) has been shown to be expressed in RGCs and in presumptive AC precursors, and to promote RGC differentiation downstream of the bHLH transcription factor Atoh7 [11]. While *Xenopus*, mouse, rat and human have one copy of *barhl1 *and *barhl2 *each, zebrafish has three *barhl *paralogs possibly due to a further genome duplication event that teleosts underwent during evolution after the split from the tetrapod lineage [12,13]. On the basis of protein sequence alignment and phylogenetic analysis, it has been suggested that two of these orthologs belong to the *barhl1 *paralog group (nominated *barhl1.1 *and *barhl1.2*) while the third belongs to the *barhl2 *group [6,12]. In contrast to mouse and *Xenopus*, all three *barhl *seem to be expressed both in the brain and in the retina [12]. In medaka fish (*Oryzias latipes*), only one *barhl *has been described so far (*olbarhl*); based on phylogenetic analysis *olbarhl *has been clustered to the *barhl1 *group of paralogs [6,12,14]. To get more insights into the evolution of *barhl *paralogs with respect to retinal differentiation we took advantage of the zebrafish and medaka model systems to perform a comprehensive comparative analysis of *barhl *gene expression as compared to the one in tetrapod. By detailed *in situ *hybridization analysis we confirmed that *barhl1.2 *and *barhl2 *are expressed in the zebrafish retina but not *barhl1.1*. A meticulous inspection of *barhl1.2 *and *barhl2 *transcript distribution indicates that while *barhl2 *appears to recapitulate the expression of its mammalian and *Xenopus *counterpart, the spatio-temporal expression pattern of *barhl1.2 *is non redundant to that of *barhl2*, suggesting that *barhl1.2 *might have evolved non redundantly with respect to *barhl2 *in the retina. Interestingly, we have detected that *barhl1.2 *shows a very early onset of expression which is highly overlapping with the expression of the *atoh7 *gene demarcating the onset of RGC genesis. Furthermore, we also describe a new *barhl *paralog in medaka which, based on protein alignment, could be assigned to the Barhl2 group. By comparing the expression of medaka *barhl1 *and *barhl2*, we found that they are both expressed in the retina, in a pattern reminiscent of zebrafish *barhl1.2 *and *barhl2 *respectively. These results combined with phylogenetic and synteny analysis suggest that *barhl *retinal expression domain is an ancestral feature that has been specifically lost in tetrapod probably due to functional redundancy following the duplication-supplementation paradigm [3,4].

## Results

### Zebrafish *barhl1.2 *and *barhl2*, but not *barhl1.1*, are expressed in distinct spatio-temporal domains of the developing retina

**Figure 1:**
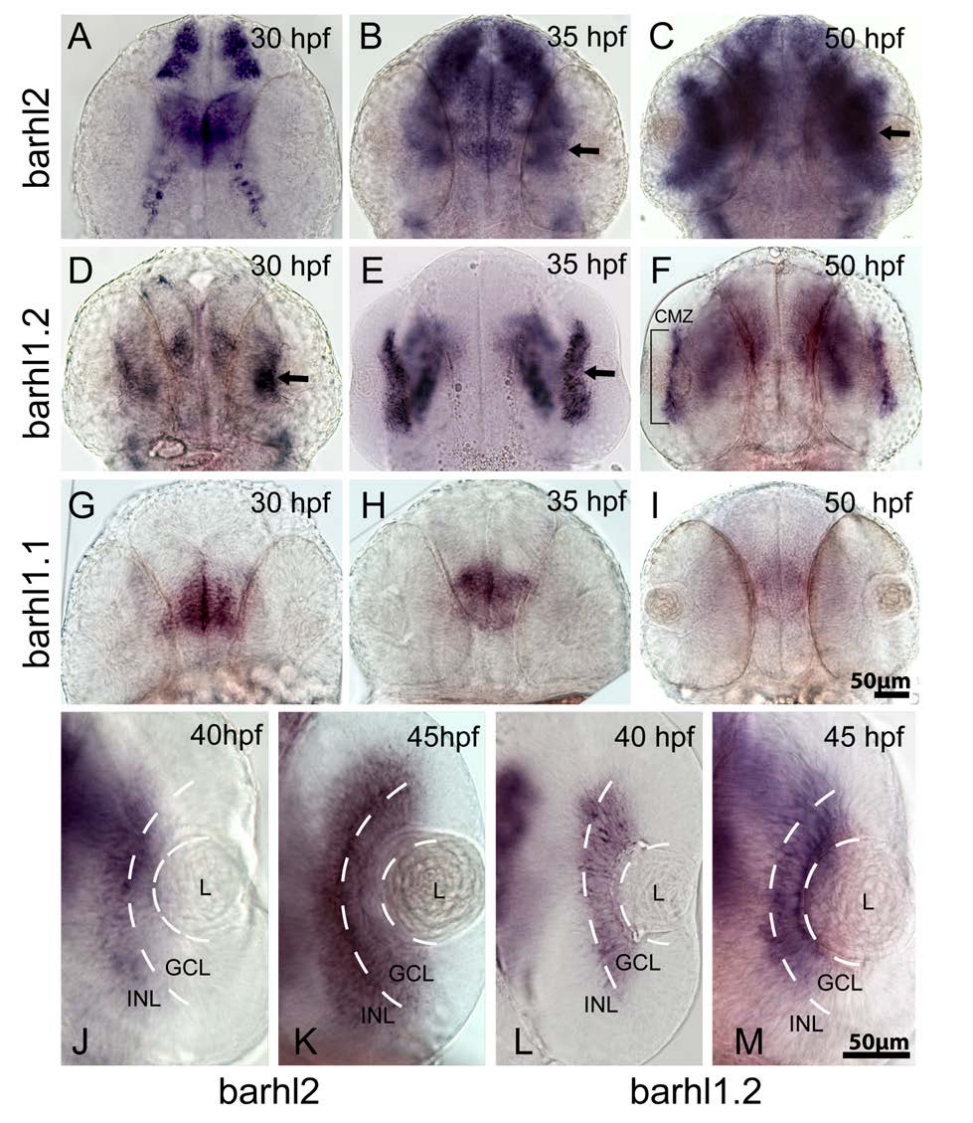
**Comparative *in situ *hybridization of *barhl *paralogs expression in the zebrafish retina**. Dorsal view of wild-type zebrafish embryos hybridized with *barhl2 *(A-C, J-K), *barhl1.2 *(D-F, L, M) and *barhl1.1 *(G-I), antisense RNA probes. Stages analyzed are indicated. Anterior is always to the top. (B, C, D and E) black arrows in indicate expression localized in the retina. In (F), the black bracket highlights *barhl1.2 *expression restricted in a thin retinal domain, which is the ciliary marginal zone (CMZ). (J-M) show a closer view on individual retinas. White dashed lines highlight the boundary between lens (L), ganglion cell layer (GCL) and inner part of the inner nuclear layer (INL), where ACs are located.

In light of the distinct expression of *barhl1 *and *barhl2 *in the tetrapod retina, we aimed at clarifying the retinal expression domains spanned by each of the three paralogs to get further insight into a possible non-redundant function of *barhl *paralogs in the zebrafish retina. We performed *in situ *hybridization on embryos at different developmental stages, starting from the beginning of retinal differentiation around 30 hours-post-fertilization (hpf), until 70 hpf. Transcripts of *barhl2 *are detected in the retina as early as 35 hpf (Figure 1B). At this stage, most RGCs and the first ACs are born [15]. *Barhl2 *transcripts are initially localized in a few cells in the central retina and subsequently expressed in the whole retina (Figure 1A-C), in a pattern matching the wave of AC genesis. At 40 hpf, expression appears mostly restricted to the inner part of the INL (Figure 1J) and later on it extends to the GCL (Figure 1C, K). By 70 hpf expression is maintained in both the INL and the RGC layer (data not shown). Interestingly, transcripts of *barhl1.2 *were detected in the retina already at 28-30 hpf (Figure 1D). This stage corresponds to the peak of RGC genesis. Similarly to *barhl2*, its expression is initially restricted to the central retina and subsequently spreads temporally and dorsally (Figure 1D, E). However, this expression appears largely restricted to the RGC layer (Figure 1E, L and 1M) and it later becomes restricted to the ciliary marginal zone (CMZ) (50 hpf, Figure 1F). In contrast, no evident expression of *barhl1.1 *was found in the retina at all stages analyzed, although transcripts could be clearly detected in the brain (Figure 1G-I). This observation confirms that zebrafish *barhl1.1 *is the ortholog of other vertebrate *barhl1*; which were never found expressed in the retina of tetrapod [7,8].

### Expression of *barhl1.2 *and *barhl2 *in the retina suggests distinct regulation by *atoh7*

**Figure 2:**
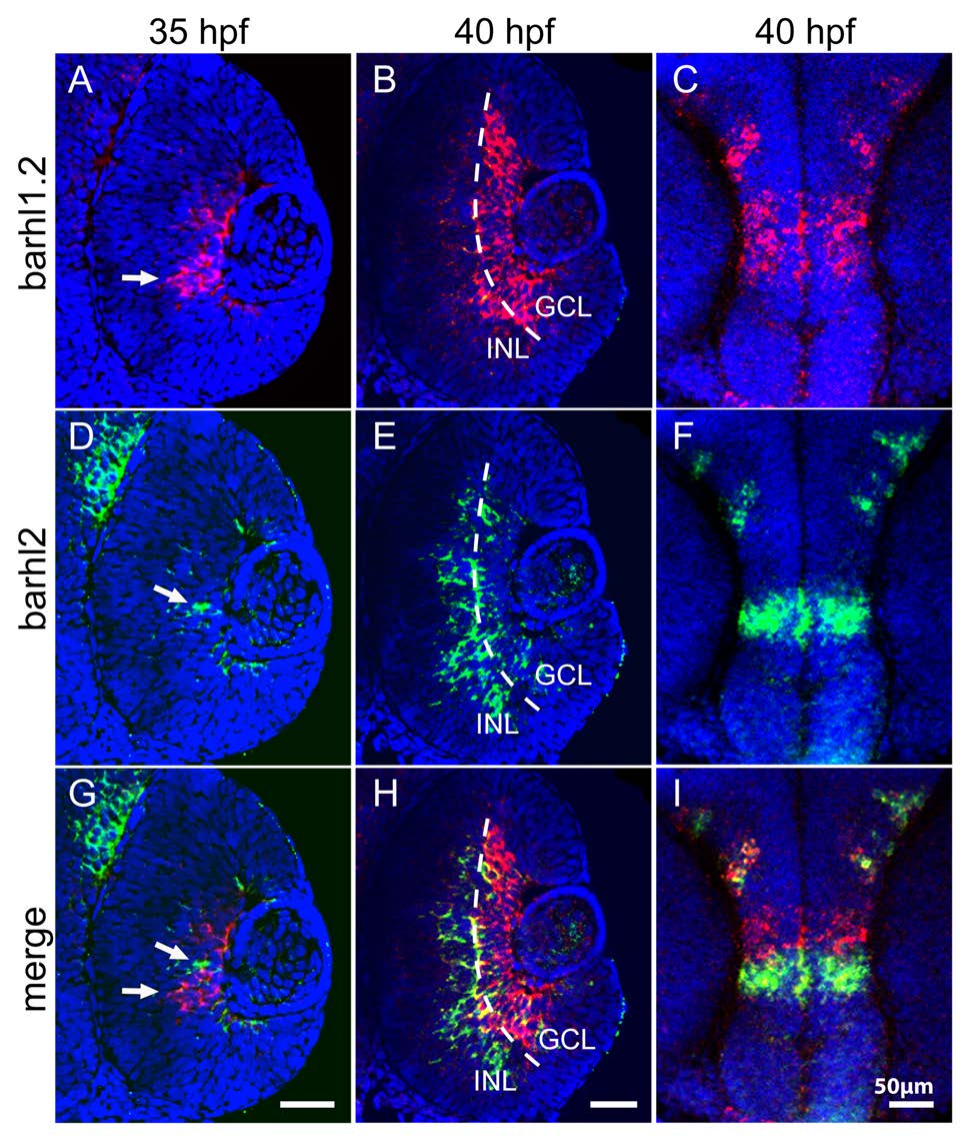
**Double fluorescent *in situ *hybridization of *barhl1.2 *and *barhl2***. Confocal sections through the central retina (A-B, D-E and G-H) or diencephalon (C, F and I) of embryos hybridized with both *barhl1.2 *and *barhl2 *antisense RNA probes. Stages analyzed are indicated. Nuclei were stained with DAPI (blue). All pictures represent a frontal view (anterior is always to the top). (A-C) *barhl1.2 *RNA antisense probe revealed with Cy3 (red). (D-F) *barhl2 *RNA antisense probe revealed with FITC (green). (G-I) green and red channel merged. White arrows in (A, D and G) indicate non-overlapping expression of the two genes. Dashed line in (B, E and H) highlights the boundary between the ganglion cell layer (GCL) and the inner nuclear layer (INL).

Given the observed non-redundant expression of *barhl2 *and *barhl1.2 *transcripts in the retina, we then investigated in greater detail the relative spatio-temporal distribution of these two transcripts by double fluorescent *in situ *hybridization (FISH). At 35 hpf, a few *barhl2*-FITC (in green) positive cells could be detected in the central retina, located within a broader domain of *barhl1.2*-Cy3 (in red) positive cells (Figure 2A, D and 2G). Within this domain, expression of *barhl1.2 *and *barhl2 *appears mostly non-overlapping (white arrows in Figure 2A, D and 2G). By 40 hpf, transcripts of *barhl1.2 *are mostly restricted to the GCL while the ones of *barhl2 *are mostly found in the inner part of the INL where ACs are present (Figure 2B, E and 2H). Interestingly, overlapping and distinct patterns of expression of *barhl1.2 *and *barhl2 *can be observed also in the brain (see example in the diencephalon in Figure 2C, F and 2I).

**Figure 3:**
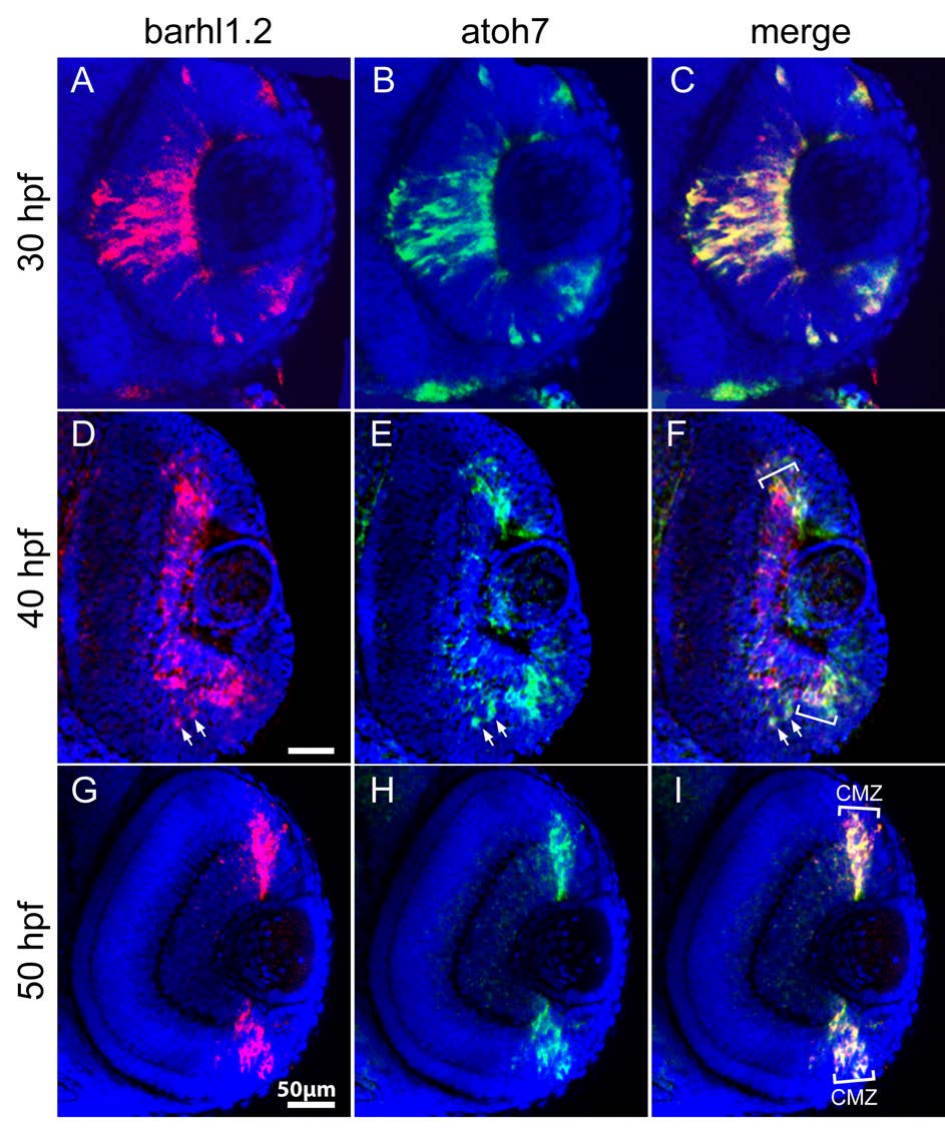
**Double fluorescent *in situ *hybridization of *barhl1.2 *and *atoh7***. Confocal sections through the central retina of embryos hybridized with *barhl1.2 *(A, D and G, in red) and *atoh7 *(B, E and H, in green) antisense RNA probes. (C, F and I) merge of red and green channels. Nuclei were stained with DAPI (blue). View is frontal in all pictures, anterior is always to the top. Stages analyzed are indicated. (D- 25 -F) Downregulation of *barhl1.2 *in the central retina is delayed with respect to the one of *atoh7 *but overlapping in the ciliary marginal zone (highlighted with white brackets CMZ). White arrows highlight two cells where both *barhl1.2 *and *atoh7 *are expressed. (F and I) the white brackets indicate the CMZ where *barhl1.2 *and *atoh7 *expression always overlap.

**Figure 4:**
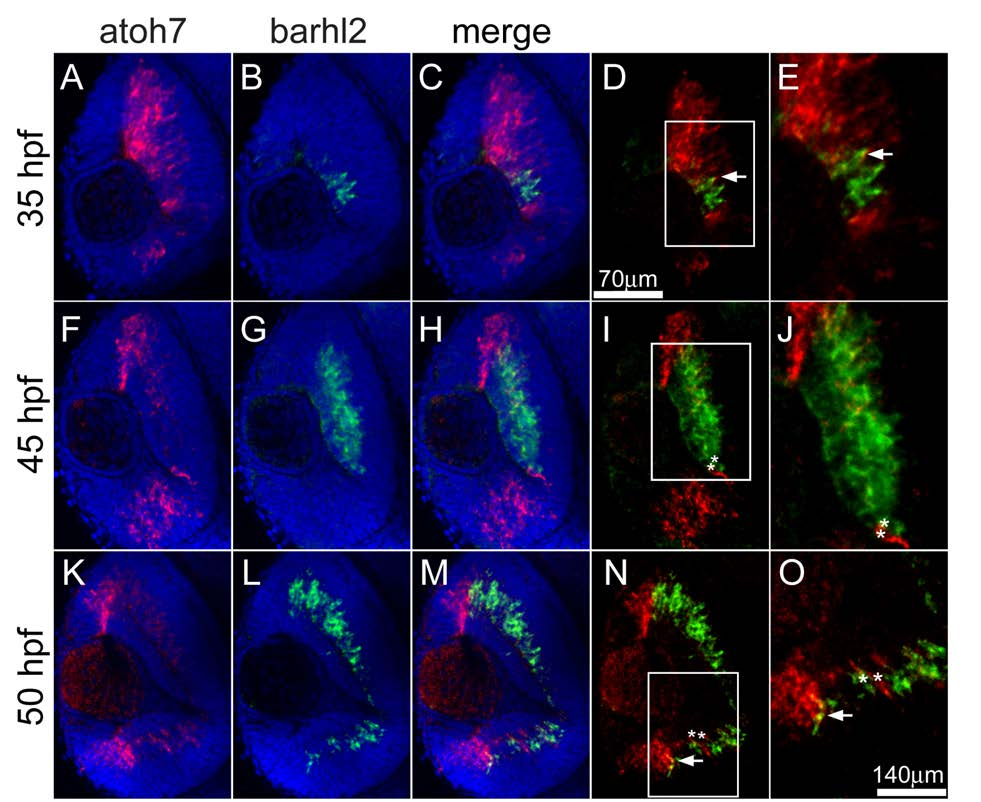
**Double fluorescent *in situ *hybridization of *barhl2 *and *atoh7***. Confocal sections through the central retina of embryos hybridized with *barhl2 *(revealed with FITC, shown in green) and *atoh7 *(revealed with Cy3, shown in red), antisense RNA probes. Nuclei were stained with DAPI (blue) to outline retinal layers. View is frontal in all pictures, anterior is always to the top. (D-E and N-O) White arrows show co-localization of both mRNAs in cells at the border of the expression domains. (I-J and N-O) white asterisks indicate adjacent cells expressing either *barhl2 *or *atoh7*. (D, I and N) white squares highlight the magnified area in E, J and O, respectively.

**Figure 5:**
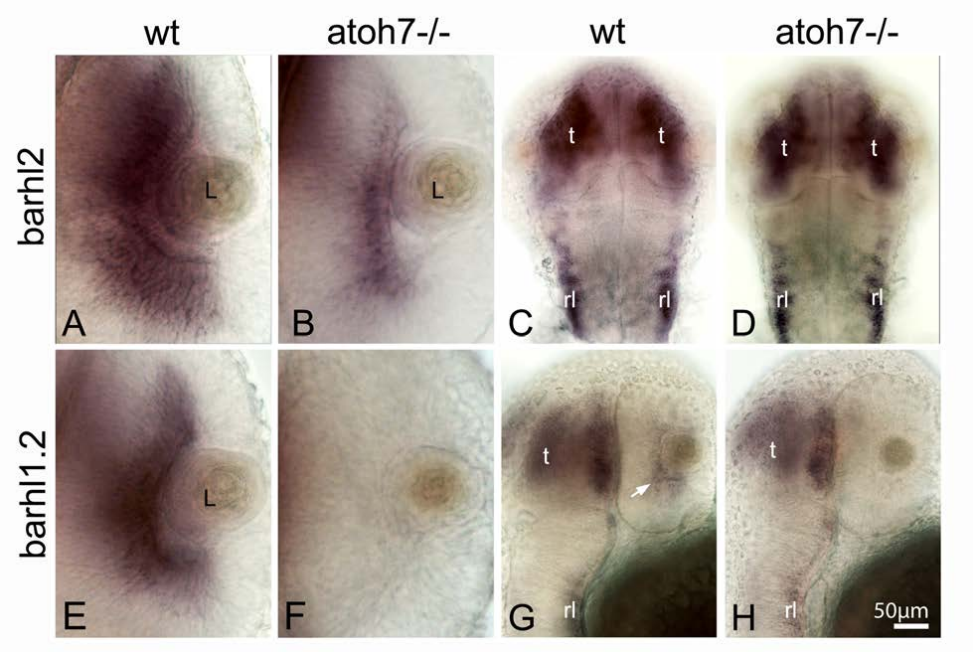
**Expression of *barhl *paralogs in the *atoh7-/- *retina**. 40 hpf zebrafish embryos hybridized with *barhl2 *(A-D) or *barhl1.2 *(E-H) RNA antisense probe. (A, E and B, F) show dorsal view of wt (A, E) and *atoh7*-/- mutant (B, F) retinas, anterior is to the top. *barhl1.2 *expression is absent in the *atoh7*-/- retina (F). (C, D) dorsal view and (G, H) lateral view at the level of the tectum (t) and rhombic lips (rl), showing that expression of both *barhl2 *(C, D) and *barhl1.2 *(G, H) remains unchanged in these brain areas. White arrow in G indicates *barhl1.2 *expression in the retina, which is missing in *atoh7*-/- mutants (H).

To map out the temporal and spatial relationship between *barhl1.2 *and *barhl2 *expression and RGC differentiation, we compared their expression to the one of *atoh7 *[16]. The proneural bHLH (basic helix-loop-helix) transcription factor Atoh7 (previously named Ath5), homolog of *Drosophila *Atonal, has been shown to be essential for RGC differentiation in mouse, zebrafish and human [17-20] and is transiently expressed in retinal precursors fated to become mainly RGCs, but also horizontal, photoreceptor and specific subpopulations of ACs [15,21,22]. FISH performed with *barhl1.2*-Cy5 **(**in red) and *atoh7*-FITC (in green) shows that at 30 hpf transcripts of both genes co-localize in the central retina (Figure 3A-C). At 40hpf, *atoh7 *transcripts start to be downregulated in the mature RGCs of the central retina (Figure 3E, F), while *barhl1.2 *expression is maintained in this area (Figure 3D-F). At this stage, we begin to observe a co-localization of *barhl1.2 *with *atoh7 *within the CMZ (Figure 3F) clearly visible at 50hpf, (Figure 3G-I). Thus, *barhl1.2 *expression overlaps and follows the expression of *atoh7 *with a slight delay, suggesting that *atoh7 *might directly or indirectly influence expression of *barhl1.2 *in progenitors adopting the RGC fate. We then analyzed the expression of *barhl2 *with respect to the one of *atoh7*. Interestingly, in this case *barhl2 *expression is mostly complementary to the one of *atoh7 *(Figure 4). At 35 hpf, *barhl2 *starts to be expressed in a central retinal domain where *atoh7 *transcripts are already downregulated (Figure 4A-E). Later on, *barhl2 *expression expands towards the peripheral retina in a fan-like wave while *atoh7 *gets restricted to the CMZ (see 45 hpf and 50 hpf in Figure 4 F-J and 4 K-O respectively). At all stages analyzed, *barhl2 *and *atoh7 *expressions appear mostly mutually exclusive (examples are highlighted with asterisks in Figure 4I-J, N-O), although few cells co-expressing both genes are always visible at the expression boundaries (as indicated by arrows in Figure 4D-E, and 4N-O). Therefore, in contrast with *barhl1.2*, the complementary expression of *barhl2 *and *atoh7 *suggests a reciprocal negative regulation between these two genes. To further investigate this aspect *in vivo*, we took the advantage of the *atoh7*-/- mutant embryos (*lakritz*). In the *atoh7*-/- retina RGCs fail to exit the cell cycle and to differentiate as a result of a loss-of-function mutation within the *atoh7 *gene [19]. We tested how *atoh7 *loss-of-function and therefore lack of RGC differentiation affect the expression of *barhl1.2 *and *barhl2 *(Figure 5). *In situ *hybridization on embryos at 40 hpf shows that *barhl1.2 *transcripts are completely missing in the GCL of the *atoh7*-/- retina (Figure 5E-F), while *barhl2 *expression is retained in this mutant retina (Figure 5A-B). The expression of both *barhl *paralogs in other brain areas, such as the tectum and the rhombic lips, is not affected by Atoh7 loss (Figure 5C-D and 5 G-H). Thus, *barhl1.2 *appears to be sustained by *atoh7 *while *barhl2 *onset of expression appears independent on *atoh7*. It remains to be demonstrated whether a negative feedback interaction exists between *barhl2 *and *atoh7*.

### Identification of a novel *barhl *paralog in medaka

**Figure 6:**
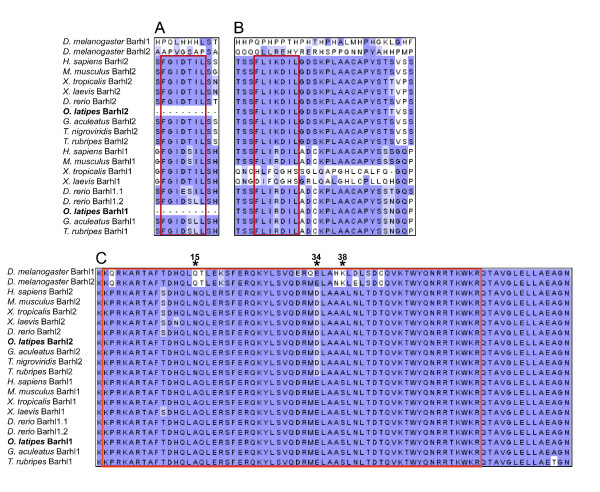
**Multiple sequence alignment of Barhl proteins**. Conserved domains found in the Barhl proteins are shown. Consensus sequences of the functional domains are in red brackets (A) FIL domain 1 is present in all vertebrates but medaka. (B) FIL domain 2 is shared between all vertebrates, but can only be partially aligned with *Xenopus*. (C) Homeodomain (conserved DNA binding motif). All Barhl2 proteins have an aspartic acid residue at position 34, an alanine instead of serine at position 38 and an asparagine residue instead of alanine at position 15 (asterisks). The blue background represents the Blosum62 score: high similarities are in dark blue while lower similarities are indicated in light blue.

**Figure 7:**
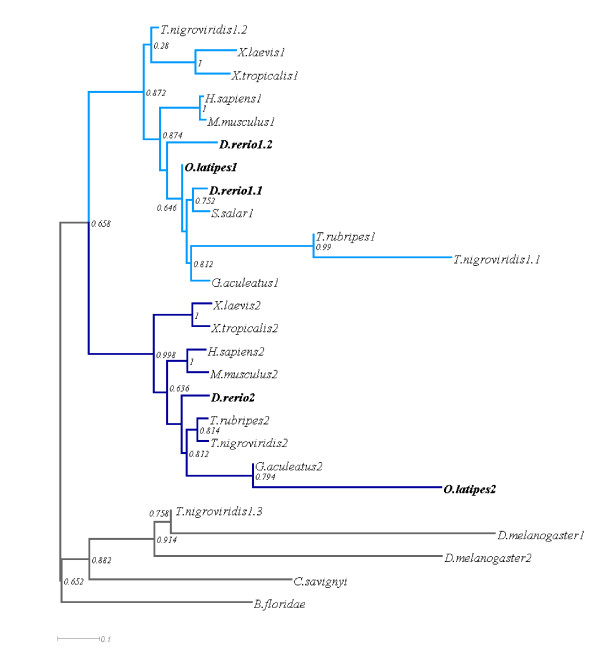
**Phylogenetic tree of Barhl homologs based on protein sequence multi-alignment**. A phylogenetic tree was inquired from the multiple sequence alignment using the neighbour-joining algorithm BioNJ. Barhl1 cluster is coloured in light blue, Barhl2 cluster in dark blue. Names related to zebrafish and medaka sequences are highlighted in bold. The numbers indicate the paralogs numbers for each species. Node values represent bootstrap support (bootstrap = 1000).

As it is commonly accepted that all teleosts underwent one further round of WGD, we wanted to test whether other teleosts have also retained more than two *barhl *and how their expression in the retina evolved. The medaka fish is a well-established model system and is therefore very suitable for a comparison with zebrafish [23]. So far, one *barhl *has been described in the medaka fish (*olbarhl*, [14]); based on phylogenetic analysis *olbarhl *has been clustered within the Barhl1 group of paralogs (*olbarhl *[6]). Interestingly, *olbarhl1 *is also expressed in the retina and in particular in the developing GCL [14]. In order to identify other putative *barhl *paralogs, we performed a BLAST search against the medaka genome (EnsEMBL Release 58, see methods) using zebrafish Barhl proteins as queries. We could identify a second member of the medaka *barhl *family on chromosome 4. By performing a multiple sequence alignment of Barhl proteins, the newly identified medaka Barhl was assigned to the Barhl2 group (Figure 6). The newly identified Barhl medaka protein has an asparagine residue instead of an alanine at position 15, an aspartic acidic residue instead of a glutamic acid at position 34 as well as an alanine instead of serine at position 38 (highlighted by asterisks in Figure 6C). The homeobox sequences of vertebrate Barhl differ at these positions and clearly divide the genes in the two mentioned groups. The FIL domains represent another set of highly conserved motifs [7,8,12,14,24]. Only one of the FIL domains, FIL2, can be found in both medaka Barhl1 and Barhl2 (Figure 6A, B). To further characterize the evolutionary origin of the new medaka ortholog, a phylogenetic tree was constructed using *Drosophila *BarH1 and BarH2 protostome outgroup (Figure 7). Additionally, we rooted the tree with Barhl protein sequences of the ancestral deuterostomes *Ciona savignyi *and *Branchiostoma floridae*, which we identified by BLAST search. *Ciona savignyi *Barhl and *Branchiostoma floridae *Barhl also cluster with the outgroup, as those two deuterostomes have not undergone the vertebrate-specific WDG and have only one ancestral Barhl protein. The Barhl proteins are clearly clustered in two groups, with the newly identified medaka sequence belonging to the Barhl2 group. Thus, medaka has one Barhl1 and one Barhl2 that relate to the homologs in other teleosts, as we would expect from the relationship among these teleost species. Our tree clearly illustrates the relationship between zebrafish Barhl1 paralogs and other teleosts Barhl1 proteins and suggests that zebrafish Barhl1.2 has split basally from the teleost Barhl1 group.

**Figure 8:**
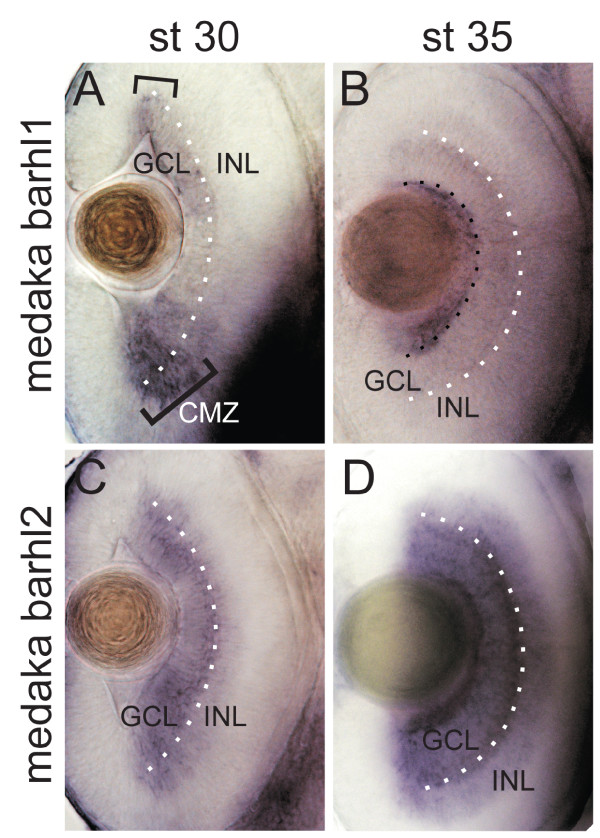
**Expression patterns of the medaka *barhl1 *and *barhl2 *in the retina**. Dorsal views through the retina of medaka embryos hybridized with either medaka *barhl1 *(A, B) or medaka *barhl2 *(C, D) RNA antisense probe. Anterior is to the top. (A) Expression of medaka *barhl1 *restricted to the ciliary marginal zone (CMZ) is highlighted with black brackets in A and with a black dotted line in B. The white dotted line indicates the ganglion cell layer (GCL)/inner nuclear layer (INL) boundary.

We then sought to compare the expression patterns of both medaka *barhl1 *and medaka *barhl2 *in the retina by *in situ *hybridization, using antisense probes against transcripts amplified from mixed stages-brain and eye cDNA (see Methods). Medaka *barhl1 *starts to be expressed in the central retina at stage 25 [14,25]. This stage more or less corresponds to 28-30 hpf in zebrafish, when *atoh7 *expression and RGC differentiation begin [23,26]. Later on, expression extends to the whole RGC layer [14]. We found that at stage 30, the medaka *barhl1 *is still expressed in the GCL but is already being downregulated in the central retina (Figure 8A). Interestingly, this is the stage at which *atoh7 *expression also starts to be downregulated in the central retina in medaka [26]. On the other hand, the medaka *barhl2 *is still strongly expressed in the central retina (Figure 8C). By stage 35, medaka *barhl1 *transcripts can be detected only in the CMZ (Figure 8B) while medaka *barhl2 *is still strongly expressed in both the GCL and the inner part of the INL (Figure 8D). Thus, the expression domain of the medaka *barhl1 *appears very similar to that of the zebrafish *barhl1.2 *as both transcripts can be found very early in the GCL and are very soon after restricted to the CMZ. Conversely, the medaka expression of *barhl2 *resembles the one observed for zebrafish *barhl2*, as both can be found strongly expressed in the INL and GCL until late stages of development.

### Conserved gene synteny between *barhl *genes

**Figure 9:**
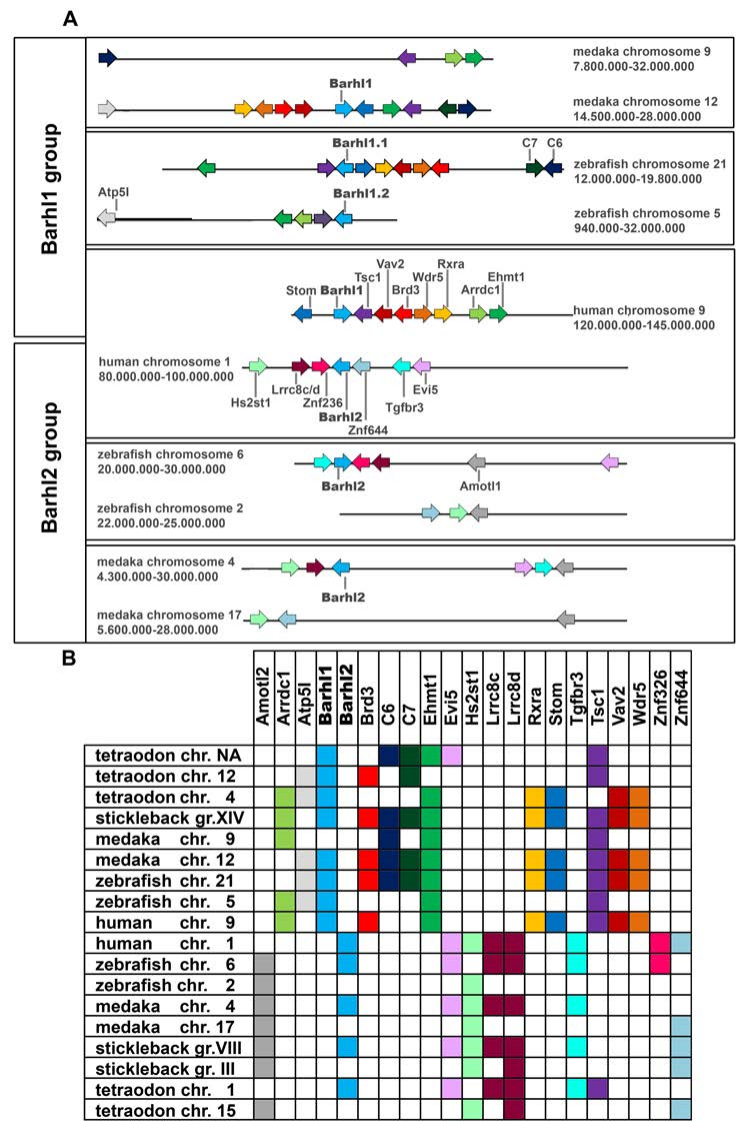
**Synteny conservation in the *barhl *locus**. (A) Chromosomal regions carrying *barhl *in human, zebrafish and medaka were compared and neighbouring genes with conserved synteny identified. Each gene is represented in a different colour as an arrow showing its orientation on the chromosome. Neighbouring genes that are in synteny are shown with a schematic indication of their distance; which is not in scale. Exact location and full name of each gene can be found in the Additional file 1:Table 1. (B) Comparison of gene distribution in the ten loci for four teleost species and human. Syntenic genes are found either in the barhl1 group or the barhl2 group.

Has the retinal expression domain been lost by one of the *barhl *duplicates, or has it been acquired after divergence of the two duplicated genes? Since the teleost branch underwent a WGD that did not affect other vertebrates, we expect to find two loci for each *barhl *gene in fish, which would explain the presence of two *barhl1 *paralogs in zebrafish, but only one paralog would have been retained in medaka. To further investigate this hypothesis, we compared the loci of *barhl *genes and looked for genes that are conserved in synteny. It has been proposed that genes that play important roles in development are surrounded by highly conserved elements and also show highly conserved gene synteny [27]. Kikuta et al. elaborated on the highly conserved synteny between the human and zebrafish barhl1 loci that might extend to the regulatory level [27]. For a more in-depth analysis we used additional teleost species to take into account syntenic conservation within these rapidly evolving fish species [28]. We searched for genes that are in synteny between zebrafish, stickleback, medaka, *Tetraodon *and human, and found that for both *barhl2 *and *barhl1*, the conservation of the locus is very high, both for non-coding sequence as well as syntenic genes. Genomic locations of all genes used in the analysis can be found in the Additional file 1: Table1. Figure 9A shows a model of the synteny relations between medaka, zebrafish and human. Human *barhl1 *is located on chromosome 9. Orthologs of the genes surrounding *barhl1 *in human can be found on zebrafish chromosomes 5 (where *barhl1.2 *is present) and 21 (where *barhl1.1 *is present) and medaka chromosomes 9 (where no *barhl *can be found) and 12 (where *barhl1 *is present). This scenario is most likely the result of WGD in teleosts and highlights that the second medaka *barhl1 *paralog, expected to be conserved in synteny on chromosome 9 has been lost during evolution. For *barhl2 *we can observe a similar pattern: The genes surrounding *barhl2 *on human chromosome 1 can be found distributed between two chromosomes each in zebrafish and medaka. Notably, in both teleost species only one duplicate of *barhl2 *has been retained. The fact that we found three or more genes that are conserved in their position as neighbours of *barhl2 *in loci that did not contain *barhl2 *(zebrafish chromosome 2, medaka chromosome 17) suggests that these chromosomal regions represent duplicated regions that did not retain a second *barhl2 *paralog. The loss of one *barhl2 *copy has been consistently observed in other teleosts species such as *Tetraodon *and fugu whereas the *barhl1 *locus seems to have undergone different genomic rearrangements. Indeed, in *Tetraodon *we found that syntenic genes around *barhl1 *are distributed to three distinct loci, whereas in stickleback, they can be found in a single linkage group (see Figure 9B for an overview on the distribution of the syntenic genes surrounding the *barhl *locus in all species considered in this analysis). Altogether, the evolution of the barhl1 locus in teleosts, coupled to WGD and species-dependent regional duplication, seems to be to be under less evolutionary constraint than in the case of barhl2, where a stereotypic pattern of gene distribution can be observed.

## Discussion

According to this study, we propose that vertebrates have two homologs of *barhl *(*barhl1 *and *barhl2*) due to WGD events that occurred before the emergence of vertebrates. This is in contrast with *Drosophila*, where two BarH genes probably arose from tandem duplication in the same locus. It is a topic of speculation whether there have been one or two rounds of WGD in early vertebrate evolution before the split of the teleosts from the other jawed vertebrates, and currently the most widely accepted view is that there were two [29,30]. Our findings are consistent with the latter view as the third paralog found in zebrafish most likely originated from the additional round of whole genome duplication that marked the rise of teleosts and did not affect other vertebrates [31]. This hypothesis is in agreement with the fact that in basal deuterostomes such as sea squirt and amphioxus, only one *barhl *locus could be found by BLAST search. From this speculation one would assume that all teleost fish once had at least three, maybe even four paralogs of *barhl*, some of which were lost. We found that in zebrafish both *barhl2 *and *barhl1.2 *are expressed in the developing retina. *Barhl1.1*, like other vertebrate *barhl1 *homologs, is not expressed in the retina whereas the medaka *barhl1 *and *barhl2 *are both expressed in the retina in a similar fashion as *barhl1.2 *and *barhl2 *in zebrafish, thus suggesting that medaka *barhl1 *has a similar dynamic of transcriptional regulation as zebrafish *barhl1.2*. Given that the *barhl *expression in the retina is an ancestral feature that was already present in *Drosophila*, the most parsimonious explanation for *barhl1 *gene evolution is that its retinal expression was maintained within the teleost lineage but was lost in *Xenopu*s and mammals. Thereafter within teleosts, the zebrafish *barhl1.1 *also lost its retinal expression probably due to a redundant function with *barhl1.2 *and relaxed evolutionary pressure in its locus. This can be further illustrated in the context of the *Tetraodon barhl1 *paralogs. These sequences do only resemble partial or split Barhl proteins and therefore the positions in the tree are excluded from the teleost barhl1 cluster. We have called the split sequences *Tetraodon *Barhl1.2 and Barhl1.3 and the protein that clusters within the teleost group Barhl1.1. In contrast, Barhl2 protein sequences are arranged in correspondence with the evolution of the species, while the retinal expression of Barhl2 has been conserved throughout the animal kingdom. This could be related to changes in function or regulation of barhl1 paralogs that led to diversification and, in some cases, retention rather than loss of a duplicate. Unfortunately, without access to expression information for *Tetraodon *and stickleback, no further hypothesis on the relationship of function and copy number can be made.

In line with these observations, our *in situ *hybridization analysis suggests that the zebrafish *barhl2 *expression pattern closely resembles that of *barhl2 *ortholog in other vertebrates, and therefore might play a role in the same neuronal lineages. The mouse Barhl2 takes part in the specification of the ACs, and later aspects of RGC maturation [9,10]. Functional experiments will be necessary to test this hypothesis in zebrafish. It also remains to be demonstrated whether the expression of *barhl2 *within the GCL is restricted to displaced ACs or RGCs or both, and whether part of *barhl2 *expression might be dependent on *atoh7*, like in other vertebrates [9]. As the expression of *atoh7 *and *barhl2 *are mostly complementary, our hypothesis is that *barhl2 *transcriptional activation in the AC lineage is independent from Atoh7. Other candidate factors might be at work in inducing *barhl2*, particularly those factors that have been shown to favour AC fate at the expense of RGCs in zebrafish [15]. The striking complementary expression of *atoh7 *and *barhl2 *that we observed (see Figure 4) would rather supports a negative feedback between the two genes. Interestingly, similar mutually excluding expression domains have been found also in Drosophila between the homolog *atonal *and *bar *genes [32]. In this study, a negative feedback has been shown to occur between the two Drosophila genes, thus suggesting the conservation of an ancestral feature. The expression of *barhl2 *in the zebrafish *atoh7-/- *retina at 40 hpf appears to be retained in time but its pattern of expression is shifted towards the lens (Figure 5B). Most ACs born at this stage become displaced in the presumptive GCL (which is devoid of RGCs, [19]); which might account for the localization of *barhl2 *expression in this domain. Thus, our current data doesn't provide, neither it excludes evidences for negative feedback between *atoh7 *and *barhl2 *in zebrafish. In contrast to *barhl1.1*, we found that *barhl1.2 *is expressed in the zebrafish retina. Surprisingly, this expression occurs nearly in synchrony with the one of *atoh7 *and the development of RGCs. Similarly, we found that the medaka *barhl1 *spatio-temporal expression is reminiscent of what has been previously described for *atoh7 *expression in the medaka retina, being first detected at stage 25 and becoming restricted to the CMZ at stage 30-35 [26]. This observation further supports the hypothesis of conserved dynamics of retinal lineages specification in both fish "twins", at least with respect to *atoh7 *and *barhl *genes. Thus, due to this extreme similarity between *barhl1.2 *and *atoh7 *expression within the RGC lineage, it is also possible that Barhl1.2 function became redundant with respect to the one of Atoh7 and was therefore lost in the tetrapod lineage. In light of this intriguing hypothesis, it will be very interesting to ask what is the retained function of the zebrafish *barhl1.2 *in the RGC lineage and in relation to *atoh7*.

After branching of the metazoan clades into protostomes and deuterostomes, a tandem replication in *Drosophila melanogaster *led to the existence of two Bar factors in the fruit fly that are assumed to be redundant. In other insects, such as *Drosophila ananassae, Anopheles *or *Apis melifera*, only one Bar can be found (results of TBLASTN search). In the line of the deuterostomes, at least one round of WGD took place before the rise of the gnathostomes (jawed vertebrates). We assume that before this period of intense genome rearrangement, there was only one Barhl, as we can find in the genome sequences of *Branchiostoma floridae *(amphioxus) or *Ciona savignyi *(sea squirt, results of TBLASTN search). Going further up in the evolutionary tree, at least two Barhls could be found in every species we looked at. These observations raise the intriguing hypothesis that Barhl factors have evolved in complexity proportional to the evolving diversity of retinal arrangement and rhabdomeric photoreceptor-derived cell types [33]. In summary, our study provides an example on how retained gene paralogs might have evolved in contributing to the specification of distinct cell-lineages in the vertebrate retina. A larger scale analysis of the functional implications of the differences in retinal key transcription factors between teleosts and other vertebrates will help to speculate further on the role of duplication retention in the evolution of vertebrates, and more specifically during eye evolution.

## Conclusions

In summary, our teleost comparison of Barhl orthologs highlights differences in expression patterns within retinal cell populations and regarding Atoh7 regulation. By extensive cross-species analysis of the *barhl *loci, these differences could be linked to differential selective pressure: while the *barhl2 *locus remains under evolutionary constraint, we show that the *barhl1 *locus rapidly evolves, thereby leading to functional differences within *barhl1 *paralogs. Our cross-species analysis provides insights on how retained gene paralogs might have evolved in relation to distinct cell-lineages in the vertebrate retina.

## Methods

### Fish lines

Breeding and rising of zebrafish followed standard protocols [34]. Zebrafish embryos were treated with 0.0045% 1-Phenyl-2-Thiourea (PTU) in medium after gastrulation to prevent pigment formation. Medaka embryos were kept in ERM medium containing 1 g/l NaCl, 30 mg/l KCl, 40 mg/l CaCl2 × 2 H2O and 163 mg/l MgSO4 × 7 H2O in deionized water.

### Ethics statement

All fish are housed in the fish facility of our laboratory, which was built according to the local animal welfare standards (Tierschutzgesetz 111, Abs. 1, Nr. 1) and in accordance with European Union animal welfare guidelines. The facility is under the supervision of the local representative of the animal welfare agency. No animal experiments were performed. Embryos of medaka (*Oryzias latipes*) and zebrafish (*Danio rerio*) were used exclusively at stages prior to hatching (not considered as animals according to German law and European union regulations). Zebrafish and medaka were raised and maintained as described previously [26]. The following strains were used for zebrafish embryos: wild type WIK/AB and for medaka embryos: the Cab wild type strain."

### Amplification and cloning of medaka Barhl2

An 800 bp fragment was polymerase chain reaction (PCR)-amplified from mixed stage medaka cDNA eye and brain using the forward primer (GAGATAGACACCGTGGGAACTGG) and reverse primer (CTGATGGAGTCCGGTACATGCTG) designed to bind in exons 1 and 4 of *olbarhl2 *(ENSORLT00000002844, EnsEMBL v58). Cycling conditions: five cycles 95°C, 10 sec, 65°C, 20 sec, 72°C, 4 min; followed by 28 cycles with annealing at 60°C. A Taq DNA-Polymerase was used for A-Tailing, incubation was for 30 min at 72°C. The PCR product was cloned into pCRII TOPO TA vector (Invitrogen) and sequenced using T7 and SP6 promoters. The sequence has been submitted to the EMBL [GenBank: JQ008931].

### Whole-mount *in situ *hybridization

Single whole-mount *in situ *hybridization of *barhl *genes was carried out as previously described in [30], for the zebrafish embryos and in [31], for the medaka embryos. Riboprobes where labelled with digoxigenin-UTP (Roche Applied Science). Hybridization with the probe was carried on over night at 65°C/68°C. Anti-DIG primary antibody coupled to alkaline phosphatase (Roche Molecular Biochemicals) and NBT-BCIP (Roche Molecular Biochemicals) was used for signal detection. For the FISH on zebrafish embryos, modifications were applied to the method described in [30], as suggested by Stephan Kirchmaier (unpublished). Embryos were washed with 100 μl Tyramide Signal Amplification (TSA) solution and incubated with FITC in TSA. Incubation with the *barhl2 *probe was for 30 minutes, incubation with the *atoh7 *probe was for 40 minutes. Embryos were then kept in the dark for all following steps. For detection and staining of the antisense probes, embryos were washed 5 × 10 min with TNT (0.1M Tris pH7.5, 0.15M NaCl, 0.1% Tween20), incubated with 1% H2O2 in TNT for 20 min and washed again 5 × 10 min. Embryos were blocked in TNB (2% DIG Block in TNT) for 1 h at RT and afterwards incubated with Anti-Digoxigenin-POD Fab fragments diluted 1:100 in TNB. For signal detection, Fluorescein (FITC), Cyanine 3 (Cy3) or Cyanine 5 (Cy5) Fluorophore Tyramide by PerkinElmer was used. Embryos were then incubated in 1 × 4',6-Diamidin-2-phenylindol (DAPI) in TNT over night at 4°C and washed several times in TNT the next day. Embryos stained with NBT/BCIP were mounted in 87% Glycerol on microscope slides and imaged with a Leica DM5000B, 10x or 20x air objectives, Leica CD500 microscope camera and Leica FireCam 1.7.1 software. Double fluorescent embryos were mounted in 100 × 15 mm glass bottom dishes in 1.5% low melting agarose. Confocal stacks were taken using the Leica SP5 confocal microscope, 20x water immersion objective and Leica Application Suite (LAS) software. FITC was excited at 488 nm by Argon laser, Cy3 by the 568 nm Helium-Neon laser, Cy5 at 633 nm by Helium-Neon and DAPI by an UV laser. Emission was sensed at 500-550 nm for FITC, 650-700 nm for Cy3, 650-800 nm for Cy5 and 400-500 nm for DAPI. Emission channels were imaged sequentially to avoid bleed-through of the two fluorescent signals. Pictures were processed using the open source software ImageJ version 1.43 and Adobe Photoshop CS3.

### Phylogenetic analysis

Protein sequences were obtained from EnsEMBL Genome Browser (v58) after using TBLASTN to perform a search for the zebrafish protein sequence of Barhl2 and Barhl1.1, respectively, against the genomic DNA of the used species to check the integrity of the annotated protein sequences. The following protein sequences were used: *Danio rerio *Barhl1.1 (Accession number ENSDARP00000016114*) Danio rerio *Barhl1.2 (Acc. No. ENSDARP00000051473) *Danio rerio *Barhl2 (Acc. No. ENSDARP00000093436) *Homo sapiens *BARHL1 (Acc. No. ENSP00000263610) *Homo sapiens *BARHL2 (Acc. No. ENSP00000359474) *Mus musculus *BARHL1 (Acc. No. ENSMUSP00000053147*) Mus musculus *BARHL2 (Acc. No. ENSMUSP00000084005) *Xenopus tropicalis *barhl1 (Acc. No. ENSXETP00000013720) *Xenopus tropicalis *barhl2 (Acc. No. ENSXETP00000051744) *Gasterosteus aculeatus *Barhl1 (ENSGACP00000023974) *Gasterosteus aculeatus *Barhl2 (ENSGACP00000005730) *Tetraodon nigroviridis *Barhl2 (ENSTNIP00000016761) *Tetraodon nigroviridis *Barhl1.1 (ENSTNIP00000008103) *Tetraodon nigroviridis *Barhl1.2 (ENSTNIP00000008179) *Tetraodon nigroviridis *Barhl1.3 (ENSTNIP00000008783) *Takifugu rubripes *Barhl1 (ENSTRUP00000013477) *Takifugu rubripes *Barhl2 (ENSTRUP00000032575) *Oryzias latipes *Barhl1 (Acc. No. ENSORLP00000015485) *Ciona savignyi *Barhl (ENSCSAVP00000019219) *Drosophila melanogaster *Bar1 (Acc. No. FBpp0074204) *Drosophila melanogaster *Bar2 (Acc. No. FBpp00742043) Sequences obtained from National Center for Biotechnology's (NCBI) GenBank database (Release 178): *Xenopus laevis *Barhl1 (Acc. No. AAG14451.1) *Xenopus laevis *Barhl2 (Acc. No. NP_001082021.1) *Salmo salar *Barhl1 (Acc. No. NP_001167081.1) *Branchiostoma floridae *Barhl1 (XP_002596391.1). The protein sequence of *Oryzias latipes *Barhl2 was obtained from translating the sequenced cDNA clone using the ExPASy online translate tool from Swiss Institute of Bioinformatics (SIB). Alignment of the sequences was produced using MUSCLE online at the European Bioinformatics Institute (EBI) [35]. A phylogenetic tree was assembled using BioNJ online at phylogeny.fr performing 1000 bootstraps and using Jones-Taylor-Thornton matrix. This distance-based algorithm is claimed to be well suited for comparison of sequences with high substitution rates [36,37]. The tree was visualized using the open source software Dendroscope [38].

### Synteny analysis

Chromosomal loci of barhl genes in human, zebrafish, stickleback, *Tetraodon *and medaka were compared by identifying all genes that occur in more than one of the loci. The position of each of these genes was then searched in all species using the EnsEMBL database search function (EnsEMBL release v58). Position and identity of genes were plotted schematically according to their order and orientation (Figure 9, not to scale). Exact position of each gene can be found in the Additional file 1: Table 1.

## Authors' contributions

LP conceived and supervised the study. LNS, LP and SA designed the experiments. LNS and SA performed and analyzed all the experiments presented. LP and LNS wrote the manuscript. MR contributed with the phylogenetic and synteny analysis, data interpretation, and to writing the manuscript. All authors revised and approved the final manuscript.

## Supplementary Material

Additional file 1 **Table 1: Genomic location of genes used for the syntenic analysis**. Genomic locations according to EnsEMBL Database release 60. The numbers indicate chromosome number:location on chromosome:strand (1 = sense, -1 = antisense). Un_random indicates sequences that have not been allocated to a specific chromosome. Click here for file
