# Supplementary material for: Evolutionary relationships and diversification of barhl genes within retinal cell lineages
Source: BMC Evol Biol. 2011 Nov 21;11:340. doi: 10.1186/1471-2148-11-340 (PMC3235082; doi:10.1186/1471-2148-11-340)
Supplement: Additional file 1 — Table 1: Genomic location of genes used for the syntenic analysis. Genomic locations according to EnsEMBL Database release 60. The numbers indicate chromosome number:location on chromosome:strand (1 = sense, -1 = antisense). Un_random indicates sequences that have not been allocated to a specific chromosome. [file 1471-2148-11-340-S1.PDF]

Additional file1

Table1 - Genomic location of genes used for the syntenic analysis

Genomic locations according to EnsEMBL Database release 60. The numbers indicate chromosome number:location on chromosome:strand (1=sense, -1=antisense). Un_random indicates sequences that have not been allocated to a specific chromosome.

Gene name	Human	Zebrafish	Medaka	Stickleback	Tetraodon
amotl2 (angyomiotine like protein 2)		6:27700245-27707602:1 and 2:22309501-22318819:1	4:28438480-28444198:-1 and 17:25395050-25400128:-1	groupIII:1714387-1718906:1 and groupVIII:1888892-1893544:1	15:5861338-5867517:1
arrdc1 (arrestine domain containing 1a)	9:140500106-140509812:1	5:31155334-31170407:-1	9:31107672-31121489:1	groupXIV:11239906-11250382:-1	4:9386747-9403198:1
atp5I (Atp synthase mitotic complex F0)	4:666225-668127:-1	5:9463872-9466910:-1_ and 21: 19,205,627-19,209,356-1	12:14578395-14580621:1		4:1367796-1368919:-1 and 12:12261324-12262671:1
barhl1 (Bar homologue-like 1)	9:135457993-135465640:1	21:15990279-15994007:-1 and 5:31384848-31388696:-1	12:23388397-23398396:-1	groupXIV:11471663-11476209:-1	12: 12,494,193-12,494,550 and 4:9,220,799-9,221,248 and Un_random: 78,595,679-78,598,276 -1
barhl2 (Bar	1:91177159-	6:21030149-	4:5541039-5543137:-1	groupVIII: 2,600,090-	1: 10,227,913-

homologue-like 2)	91182794:-1	21033975:1		2,602,935	10,230,408 -1
brd3 (bromodomain containing protein 3)		21:16887342-16895702:1	12:22216867-22225874:1	groupXIV:12011314-12017087:-1	12:12471415-12475065:-1
c6 (complement component 6)	5:41142336-41261540:-1	21:19743335-19760478:-1	12:27464701-27480125:-1	groupXIV:10371247-10378853:1	Un_random:48782021-48783553:1
c7 (complement component 7)	5:40909354-40983041:1	21:19729381-19745546:1	12:27434700-27462812:1 and 9:7832925-7839222:1	groupXIII:5768682-5775531:1 and groupXIV:10379024-10386421:-1	Un_random: 49766054-49767590:1 and Un_random: 74030120-74033807:-1 and 12:8686203-8697279:-1
ehmt1 (histone lysine N methyl transferase)	9:140513444-140764468:1	21:12846348-12894216:-1	12:25500672-25521327:1 and 9:31132057-31147203:1	groupXIV: 10,826,287-10,835,393 1	Un_random:48208321-48209363:-1 and 4:9,683,349-9,690,500 -1
evi5 (viral integration site 5)	1:92974253-93257961:-1	6:28725747-28800269:-1	4:25380101-25406765:1	groupVIII:2356295-2370131:1	1:10442745-10459360:-1
hs2st1 (heperan sulfat sulfur transferase)	1:87380331-87634887:1	2:23147821-23181294:-1	17:5714247-5734728:1 and 4:5010500-5028634:1	groupVIII:2924350-2930528:-1 and groupIII:8958002-8973430:-1	Un_random: 81851403-81854671:1 and 15:3646954-3657977:1
lrrc8c (leucin rich	1:90098631-	6:21400489-	4:5325826-5342313:1	groupVIII:2737798-	1:10111297-

repeat containing 8c)	90398809:1	21414270:-1		2741487:-1	10114872:1
lrrc8d (leucin rich repeat containing 8d)	1:90286604-90401982:1	6:21383889-21393511:-1	4:5347870-5350470:1	groupVIII:2731382-2733913:-1 and groupIII:8919560-8922106:-1	15:3680581-3683127:1 and 1:10117991-10120525:1
rxra (retinoic acid receptor rxr alpha a)	9:137208944-137332431:1	21:16279939-16459148:1	12:22162888-22205938:1	groupXIV:12026280-12052674:-1	4:8807963-8829940:1
stom (erythrocyte band 7 integral membrane protein)	9:124101355-124132531:-1	21:16054176-16078983:1	12:23339705-23349787:-1	groupXIV: 11,510,570-11,516,686 1	4:9185829-9191347:-1
tgfbr3 (transforming growth factor beta receptor III)	1:92145900-92371559:-1	6:20632844-20810939:1	4:25542773-25585566:1	groupVIII:2470284-2502839:1	1:10349367-10374528:-1
tsc1 (tuberos sklerosis 1)	9:135766735-135820020:-1	21:15818677-15849738:1 and 5:31291004-31305899:1	12:26907551-26935182:-1 and 9:27141512-27143868:-1	groupXIV: 11,383,328-11,397,058 1	1:10349367-10374528:-1 and 12:12463369-12467464:1 and Un_random:49032301-49033650:-1 and Un_random:49035583-49036931:1

vav2 (vav2 guanine nucleotide exchange factor)	9:136627016-136857726:-1	21:16536263-16581224:-1	12:22448925-22482493:1	groupXIV: 11,850,201-11,893,984 -1	4:8908162-8938285:1
wdr5 (wd repeat containing protein 5)	9:137001210-137025093:1	21:16871613-16886576:-1	12:22209489-22216544:-1	groupXIV:12018544-12022995:1	4:8831270-8833463:-1
znf326 (zinc finger protein 326)	1:90457722-90494097:1	6:21370407-21378273:-1			
znf644 (zinc finger protein 644)	1:91380859-91487829	2:23021858-23039201:1	17:5831104-5838891:-1	groupVIII:2537139-2541472:1 and groupIII:8906145-8912085:1	15:3688484-3693319:-1
